# An audit of the quality of online immunisation information available to Australian parents

**DOI:** 10.1186/s12889-016-3933-9

**Published:** 2017-01-13

**Authors:** K. E. Wiley, M. Steffens, N. Berry, J. Leask

**Affiliations:** 1 National Centre for Immunisation Research and Surveillance, The Children’s Hospital at Westmead, Sydney, NSW 2145 Australia; 2 Sydney School of Public Health, University of Sydney, Sydney, NSW 2006 Australia; 3 Sydney Nursing School, University of Sydney, Sydney, NSW 2006 Australia

**Keywords:** Immunisation, Health information, Internet search, Online information quality

## Abstract

**Background:**

The Internet is increasingly a source of health information for parents, who use the Internet alongside health care providers for immunisation information. Concerns have been raised about the reliability of online immunisation information, however to date there has been no audit of the quality or quantity of what is available to Australian parents. The objective of this study was to address this gap by simulating a general online search for immunisation information, and assessing the quality and quantity of the web sites returned by the search.

**Methods:**

We used Google trends to identify the most common immunisation search terms used in Australia. The ten most common terms were entered into five search engines and the first ten non-commercial results from each search collated. A quality assessment tool was developed using the World Health Organization Global Advisory Committee on Vaccine Safety (GACVS) criteria for assessing the quality of vaccine safety web sites, and used to assess and score the quality of the sites.

**Results:**

Seven hundred web pages were identified, of which 514 were duplicates, leaving 186 pages from 115 web sites which were audited. Forty sites did not include human immunisation information, or presented personal opinion about individuals, and were not scored. Of the 75 sites quality scored, 65 (87%) were supportive of immunisation, while 10 (13%) were not supportive. The overall mean quality score was 57/100 (range 14/100 to 92/100). When stratified by pro and anti-vaccination stance, the average quality score for pro-vaccine sites was 61/100, while the average score for anti-vaccine sites was 30/100.

Pro-vaccine information could be divided into three content groups: generalist overview with little detail; well-articulated and understandable detail; and lengthy and highly technical explanations. The main area found to be lacking in pro-vaccine sites was lack of transparent authorship.

**Conclusion:**

Our findings suggest a need for information which is easily found, transparently authored, well-referenced, and written in a way that is easily understood.

## Background

The internet has changed the way people find health information [1]. Recent figures show that, of the 13.3 million Australians who access the Internet at home, research activities were among the most commonly reported undertakings [2]. Increasingly, parents are using Internet-sourced information in addition to traditional consultations with health care providers to improve their understanding of children’s health-related issues [3]. One study estimates 43% of Australian parents access child health information on the Internet [4].

Negative online messages are thought to undermine parents’ confidence in vaccines [5], leading government and health care providers to raise concerns about the reliability of health information available on the internet. However, no comprehensive assessment of the quantity or quality of online general immunisation information available to Australian parents has been conducted. A previous study by Wolfe and Sharp assessed the pro- or anti-vaccine stance of parental internet search results for immunisation information overseas [6] and found that the search terms and search engine used had a bearing on the amount of pro- or anti- vaccination material returned by the search, and the order in which they were presented. Another study by Sak et al. compared the quality of pro- and anti-vaccine web sites, but the sample was not intended to be representative of what a typical parent would see when searching [7]. Finally, a previous unpublished study examined online information available in 2007 [8]. This study used only three search terms (vaccine, vaccination and immunisation), and various iterations of one search engine (http://www.google.com, http://www.google.com.au, http://www.google.co.uk, http://www.google.ie, www.google.co.za and www.google.co.nz). The results were tabulated and assessed as “pro- or anti-vaccine”, and the intended audiences given (parents or health care workers). None of these studies sought to assess both the quality and quantity of the information given by a web search undertaken by a typical parent.

This study aims to address this gap in knowledge by investigating the quantity and quality of general immunisation information available to Australian parents who search for it online.

## Methods

### Search strategy

We identified the immunisation themed search terms most commonly used by Australians using Google Trends [9]. We compared the relative frequencies with which the terms “vaccine”, “vaccination”, “immunisation”, “immunization” and “childhood vaccines” occurred in Australian internet searches between 2007 and 2014 in order to design a search strategy that closely simulated Australian parents’ online search behaviour. In Australia, the terms ‘vaccine’, ‘vaccination’ and ‘immunisation’ occurred relatively frequently. We used the ‘related search terms’ function to identify search terms that most commonly occurred alongside these three search terms. As the scope of this study was to examine the quantity and quality of general vaccine information, search terms relating to specific vaccines (for example, “flu vaccine” or “whooping cough vaccine”) were removed from this list. The reasons for this decision were twofold: Firstly, research shows that the majority of Australian parents are “unquestioning acceptors” (30–40%) or “cautious acceptors” (25–35%) of vaccines [10]. We therefore modelled our search on the general information this majority would look for on line in the first instance. Secondly, while we acknowledge that some hesitant parents may have concerns about specific vaccines, the majority of concerns held by parents are generally applicable across all vaccines [11], and therefore would be covered by a more general search. Furthermore, time and resource constraints meant that we could not include all of the individual vaccines on the Australian childhood schedule in our search terms. The resulting list of search terms is presented in Table 1.

**Table 1:** List of search terms entered into search engines to assess what information is returned when Australian healthcare consumers search for generic immunisation information

Primary generic search term	List of related search terms returned by Google Trends	Final selected list of generic search terms examined
Vaccine	Vaccination	Vaccine
	Vaccine side effects	Vaccine side effects
Vaccination	Vaccination Australia	Vaccination
	Vaccine	Vaccination Australia
	Vaccination schedule	Vaccination schedule
	Vaccinations	Australian Vaccination
	Australian Vaccination	Immunisation
	Immunisation	Immunisation schedule
Immunisation	Immunisation schedule	Immunisation Australia
	Immunisation Australia	Australian Immunisation
	Australian Immunisation	Immunisation Handbook
	Immunisation Handbook	Child immunisation
	Child immunisation	Childhood immunisation
	Vaccination	Baby immunisation
	Childhood immunisation	
	Baby immunisation	

We identified the most commonly used search engines based on a previous unpublished study [8] and the Search Engine List (an on-line resource reporting this information) [12]. The search engines used were: Google.com.au; bing.com; yahoo.com; Ask.com; and Lycos.com.au. When options of searching the entire internet or Australian sites only were offered by the respective search engines, the Australian option was selected.

In order to minimise ‘filter bubble’ effect (the automatic personalisation of the search results based on previous browsing history), the researcher cleared the browser history prior to conducting the searches and did not sign in where this option was offered [13]. All searches were conducted on a single day, March 5 2014. The final list of terms in Table 1 were entered, one at a time, into each of the five search engines listed above. The top ten results of each search were copied into a separate document as a record of the search.

The top ten web pages returned for each search term/search engine combination were catalogued in a Microsoft Excel spread sheet and duplicate entries removed. Sponsored and advertised sites were not included, as research has shown people conducting a targeted search, such as for immunisation information, tend to avoid advertising material [14, 15]. Web sites that did not include information about childhood vaccination, such as news listings and personal web pages were also excluded.

**Fig. 1:**
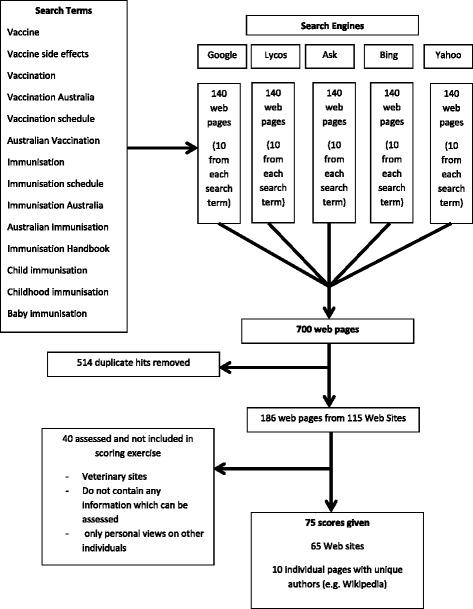
Search flow diagram

The web pages were then used as “entry points” to the broader web site on which they were found, and the web site assessed as a whole. Wikipedia and publicly available Facebook pages were assessed individually, as each page is written and edited by different groups or individuals (See Fig. 1).

### Quality assessment

To help guide people in how to identify good quality immunisation information online, the World Health Organization (WHO) Global Advisory Committee on Vaccine Safety (GACVS) developed “Good information practices for vaccine safety web sites” to “assist readers of websites in identifying those sites providing information on vaccine safety that comply with good information practices” [16]. This resource includes a checklist of attributes a reader can look for to aid in their assessment of the quality of the web site.

We developed a 43-item quality assessment tool based on the WHO GACVS checklist criteria for assessing the quality of vaccine safety web sites which is divided into six thematic domains: Mission of site; Disclosure of ownership; Transparency of sponsorship; Accountability to users; Quality of information; and Quantity of information (See Table 2). Items in the checklist were not universally applicable to all of the websites under consideration. Therefore, each item was scored as present (1), absent (0), or not applicable. The total number of not applicable items was subtracted from the denominator (43) to give an adjusted denominator, the highest possible score for that site. A score out of 100 was then calculated by expressing the total number of attributes present divided by the adjusted denominator as a percentage (see Table 2). The web pages included in the audit were divided into two groups and assessed independently by two researchers who met weekly to resolve uncertainty to ensure consistency was maintained. The final score was made by consensus between the two researchers (KW and MS).

**Table 2:** Web site quality score criteria

Domain	Item number	WHO GAVCS Attribute
1. Mission of site	1	Purpose of the website stated.
	2	Intended audience defined.
	3	Site provides a mission statement.
	4	The content of the site matches statement.
2. Disclosure of ownership/source	5	Individual or organization name on every page?
	6	Physical address on every page?
	7	Electronic address on every page?
	8	Qualifications/credentials of organization or individual site owner specified
	9	Type of organization made clear (e.g., government, non-profit, commercial)
	10	Organization or individual's affiliations and alliances and disclosure of any relationship that might influence the content of the site.
	11	Editorial Board, Advisory Board, or Board of Directors members listed with credentials.
3. Transparency of sponsorship	12	Disclosure of all sources of funding for organization/website (grants, sponsors, advertisers, fees, personal).
	13	Disclosure of any relevant personal or financial associations that might be considered a potential conflict of interest.
	14	If advertising is a source of funding, this should be clearly stated
	15	A brief description of the owner's advertising policy is included.
	16	Content intended to promote or sell a product or service should be clearly distinguished from the educational and scientific content.
4. Accountability to Users	17	Multiple methods of contacting the owner of the site (e-mail address, electronic form, mail, phone, fax) must be available from the home page.
	18	Multiple methods of contacting the owner of the site (e-mail address, electronic form, mail, phone, fax) must be easily accessible from other pages of the site.
	19	A site offering interactive exchanges (e.g., chat room, medical advice) provides information about the moderator or clinician's expertise and affiliations.
	20	A site offering interactive exchanges (e.g., chat room, medical advice) provides information about the moderator or clinician's source of compensation
	21	A site offering interactive exchanges (e.g., chat room, medical advice) provides a disclaimer that all posted information may not be accurate.
5. Quality of information	22	Authority of sources: Clear statement of source for all information, including 1author's name
	23	Authority of sources: Clear statement on the sources of information, including Author's credentials and / or affiliations
	24	Authority of sources: Clear statement on the sources of information, including Author's financial disclosure or potential conflict of interest
	25	Description of any “seal of approval” or award the website has been granted.
	26	Attribution: All information supported by citations to source resources with hypertext links if available.
	27	The site should indicate whether information is based on scientific studies, expert consensus, professional opinion, or personal experience or opinion.
	28	Accuracy: The information presented should be based on objective, scientific research.
	29	The site should identify the evidence that supports a position including references to published studies and reference works.
	30	Currency: The date that content was first developed.
	31	The date of last update or modification should be clearly indicated on each piece. The date the whole site was updated or the copyright date is not adequate.
	32	Site does not contain out-dated information
	33	Review process: Statement of procedure used for selection of site content.
	34	Includes a guarantee of the independence of the editorial process.
	35	Includes the names and credentials of the Editorial Board.
	36	Standards of writing/editing: Writing on the site should be professional, with proper grammar, spelling, and composition.
	37	Completeness: Includes the comprehensiveness of a resource, including the breadth and depth of coverage.
6. Quantity of information	38	Includes the retrospective coverage (archived items).
	39	Includes the balance of the information presented, such as admitting when an issue is controversial and including all reasonable sides in a fair way.
	40	Provision of links to other resources: Offers hypertext links to other resources.
	41	Indicate whether links to other sites are informational only or if such links imply endorsement.
	42	Any links are carefully selected and their content is accurate and credible.
	43	Any links are carefully selected and their content is current.

Web sites which placed a lot of emphasis on the dangers of vaccines, expressed doubt about their necessity, or gave advice that vaccines should be avoided were classed as “not supportive of vaccines”. Web sites which placed emphasis on the positive aspects of vaccines, and recommended their use were classed as “supportive of vaccines”.

The author’s informed subjective observations on the health literacy accessibility of pro-vaccine sites were then discussed, with a focus on how well the information would meet the needs of parents according to their position on vaccination as defined by Leask et al. [10].

## Results

### Quantity of information

Seven hundred web pages were returned by the combined searches, of which, 514 were duplicates, leaving a total of 186 pages from 115 web sites. (Refer to Fig. 1). Of these, 40 web sites were found not to include immunisation information, referred to veterinary vaccines, or presented personal opinion about people involved in immunisation, and were therefore excluded, leaving 65 web sites and ten individual pages included in the quality scoring exercise. The ten individual pages were from sites such as Wikipedia where individual pages have different authors, and therefore required scoring separately.

Some sites were represented more often than others in the search results, because a number of the pages returned originated from the same site. Table 3 lists the 11 web sites for which more than four pages were returned by our search. The most commonly occurring web site was the American Centres for Disease Control and Prevention, followed by the Australian Government Department of Health’s Immunise Australia website, and various other government and independent research bodies.

**Table 3:** Web Sites for which more than one page was identified by the search

Web Site Title	Web site address	Number of unique pages identified by the search
Centres for Disease Control and Prevention (USA)	http://www.cdc.gov	12
Immunise Australia Program (Australian Federal Government Department of Health)	http://www.immunise.health.gov.au	7
Australian Government Department of Health	http://www.health.gov.au	6
National Centre for Immunisation Research and Surveillance (Australia)	http://www.ncirs.edu.au	6
Wikipedia (US)	http://en.wikipedia.org	5
New South Wales Health (NSW Government, Australia)	http://www.health.nsw.gov.au	5

### Pro- and anti-vaccination stance

Of the total 75 web sites quality assessed, 65 (87%) were supportive of immunisation, while 10 (13%) were not supportive. To gain perspective on what parents saw at the top of their search results, we analysed the first web page returned by each search. This analysis revealed that of these 70 “top hits” 64 (91%) were supportive of immunisation. The remaining 6 pages (8%) were not supportive of vaccination, most of which were published by the Australian Vaccination Sceptics Network (the main anti-vaccine lobby group in Australia). When these “top hits” were viewed by search engine, Yahoo returned the highest number of pages which were not supportive of vaccination across the search terms, with three hits (all from the Australian Vaccination Sceptics Network). Google followed with two hits (both also from the Australian Vaccination Sceptics Network), while ASK returned one hit for a book on Amazon which offered alternatives to vaccination. Neither Lycos nor Bing returned pages not supportive of vaccination as a first hit for any of the search terms.

### Origin of web sites

Of the final 75 pages scored originating from 65 unique websites, 42 were hosted in Australia, 25 in the United States, six in the United Kingdom, and one each in Ireland and Switzerland.

### Quality of information

The overall scores indicate that the quality of vaccine information the web sites contained was variable. The mean overall quality score across all 75 scored web pages was 57/100, with a maximum of 92/100 (www.historyofvaccines.org), and a minimum of 14/100 (www.vaccine-side-effects.com) (Table 4).

**Table 4:** Overall mean quality score, and mean domain scores of audited web sites on immunisation

	Mean standardised quality score* of individual domains of scoring system						Overall standardised score** (n/100)
Domain:	1. Mission of Site (n/100)	2. Disclosure of ownership (n/100)	3. Transparency of Sponsorship (n/100)	4. Accountability to users (n/100)	5. Quality of information (n/100)	6. Quantity of information (n/100)	
Mean:	73	57	57	72	53	58	58

*Standardised domain quality score calculated as: (total number of items present)/(total number of items in domain – total number of items not applicable in that domain for that web site) × 100

**Overall standardised score calculated as: (total number of items present)/(43 – total number of items not applicable for that web site) × 100

When stratified by pro and anti-vaccination stance, the average overall score for sites which were “supportive of vaccines” was 61/100, while the average score for sites “not supportive of vaccines” was 30/100.

**Fig. 2:**
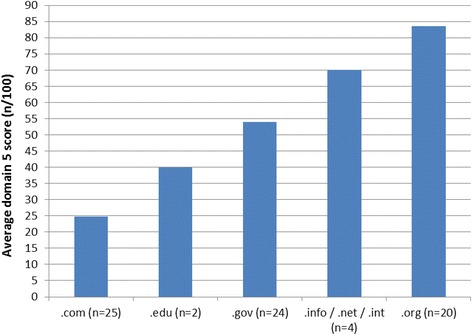
Comparison of quality domain scores (domain 5 of the quality assessment tool) among internet domain types

When the total quality scores were disaggregated into the individual domain scores (refer to Table 4), it became evident that on average, the sites were clear in their mission, and the content generally matched the stated mission (domain 1). It can also be seen that, on average, the sites scored relatively well for accountability to users (domain 4), providing adequate contact details for the site owner, and where interactive content was available, the appropriate disclaimers and declarations were given. The three domains with lower average scores were disclosure of ownership (domain 2), transparency of sponsorship (domain 3) and quality of information (domain 5). At an individual level, many sites were not clear on ownership or funding sources. From a quality perspective, many sites contained correct information; however they often did not go further than general statements such as “Immunisation is a simple, safe and effective way of protecting your child against harmful diseases that can cause serious health problems and sometimes death” [17]. Even when more detail was given, it was often not well referenced (if at all), and where references were available, the published papers themselves often required payment or subscription, and thus were not readily accessible to the general public. Similarly, the identity of the author of the information was rarely given, nor were the qualifications of the author(s) or editorial board, where applicable. When the domain 5 (quality of information) scores are stratified into internet domain types (.co/.com,.gov,.edu,.org and.net/.int/.info),.org scored the highest, followed by.info/.net/.int,.edu, and.gov, while.com internet domains scored the lowest on average in this regard (refer to Fig. 2).

### Understandability of the information for the general public

The authors’ general observations were that the pro-vaccine information returned by this search can be qualitatively divided into three broad content groups; those that gave a generalist overview without much detail; those that provided well-articulated and understandable detail; and those that gave lengthy and highly technical explanations.

The majority of sites appeared to belong to the first group. While the information was almost always technically correct, there was rarely any explanatory depth to the content. These sites typically contained unsupported statements such as “immunisation is safe and effective”. Many government web sites contained this level of information, as reflected in the comparatively lower average domain 5, “quality of information” scores for.gov internet domain types reported above. Sometimes these sites gave links to external sites, some of which fell into the second group, and (usually) the third.

The second group of sites was relatively small, and usually took the form of “fact sheets” that were often difficult to locate on the site. These sites contained technical information that was well-communicated, and more often referenced other sources than the first group, however they were rarely attributable to an author.

The third group of sites was also relatively small. They were often highly technical and written at a level suitable for someone with higher education in science or medicine. The information contained on these sites was often well referenced, usually citing peer-reviewed scientific publications; however access to these publications was, at best, limited.

## Discussion

To our knowledge this is the first published audit of general immunisation information available to Australian parents. This information will help inform the improvement of current resources, and the development of future web-based information for Australian parents seeking immunisation information.

Despite recent calls to move away from it [18], many still ascribe to the “deficit model” of immunisation information communication; it is assumed that parents who are hesitant about or refuse vaccinations for their children must not know or understand the science supporting it. Further to this, there is often a generally held perception that anti-vaccination messages are rife on line, and that parents searching online for information will be “lead astray” by this misinformation. Our findings suggest that when an unbiased search for information is undertaken, the majority (87%) of web sites are supportive of vaccination, suggesting that the volume of “pro” versus “anti” immunisation information is more in favour of “pro” than often assumed. However, most people don’t clear their browser history to ensure their web search is unbiased: Filter bubble occurs when an algorithm is used by the search engine, such as Google’s Personalized Search function, to return hits based not only on the relevance of the web site, but on the sites the user (or someone using the same browser) has visited previously. This will result in the search returning web sites that confirm or agree with the user’s previously browsed pages, potentially filtering out information that disagrees with what the user is likely to read. One study found that search results are altered if the browser history has not been cleared prior to searching by an estimated 11.7% [13]. This phenomenon would only fuel what is normal human confirmation bias (the tendency to only read what confirms one’s beliefs to begin with). Similarly, google searches include an “autocomplete” function, whereby google suggests completed search phrases based on what is typed initially. These suggestions are predicted using what is currently being typed, previous searches the user has made (if logged into their google account), and what other people are searching for [19]. This may also have an effect on what people are accessing. Possible ways to combat this is to communicate to parents (particularly those wanting “balanced” or unbiased information) to clear the browser history before undertaking a search for immunisation information, and not to use any suggested search terms.

From a quality perspective, most pro-vaccine sites contained the correct information, but it was often not well referenced (if at all), did not disclose by whom it was written, and did not give the qualifications of the author(s) or editorial board, where applicable. The majority of these sites did not go on to explain the information to any great depth, and while a relatively smaller number did go into more detail, it was either difficult to locate and not well attributed, or was very “science-heavy” and difficult to read.

**Fig. 3:**
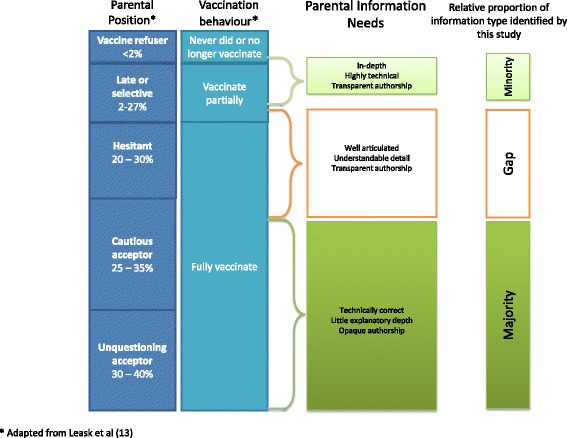
Parental positions, information needs and online information availability as identified by this search

It is widely recognised that parental vaccine acceptance is not a dichotomous schema of “acceptors” and “rejecters”, but a spectrum of acceptance behaviours [20]. Using the Vaccine Communication Framework devised by Leask and colleagues [10] and informed by Benin et al. [21], the majority of parents can be classified as “unquestioning acceptors”. These people often don’t require information beyond that supplied by the majority of sites which were technically correct, without much explanatory depth. Other parents, the ones who fall between “cautious acceptors” and “hesitant late or selective” acceptors do require more information [10]. Our findings suggest that, while these parents are arguably the ones with the greatest information needs, their needs may not be being met by what is currently available to them; the information is either too basic, or too technical, with little readily available between the two extremes (refer to Fig. 3). Our findings suggest that greater focus is warranted on developing resources that are tailored to these parent’s information needs, and include well-communicated, well-referenced and transparently authored information which is easily identified in an online search.

Finally, the information was often not easily identified on the web sites, with the immunisation information often “buried” among other components of the site. Thus, while the quality of the information contained on a given site is important, it is equally important that the information be easily found. This leads to a broader consideration of the “usability” of the information; the way in which the internet is used to communicate vaccine information is evolving. While in the past the web functioned primarily as information provision, this role has developed into a platform that is much more interactive, where user participation dictates the type of information needed and how it is used [1]. In addition to being easily found, immunisation information now needs to be more interactive.

This study has limitations. The internet is not static, and search results will vary with time, as content is added, removed, or updated. These searches were undertaken in 2014, therefore the content of some of the assessed sites may have been updated, or there may now be other information available. For example, the GACVS criteria used to develop the scoring system was updated in September 2015, after we had completed scoring the sites identified in our search. As previously mentioned, this search can only be taken as indicative of one undertaken with a cleared browser history and any personalisation of the search engine disabled. Parents searching for information while signed into their Google account for example, will be subject to filter bubble, and will therefore come across a greater volume of information aligned with their previous searches. Finally, vaccine-specific search terms were not included in this study. Excluding these terms may have resulted in some web sites not being included in the assessment, however it is likely that the general search we conducted may have identified at least some of the same sites a more specific search would have. Future studies could be considered to assess more specific search terms. Our search, however does provide a useful snapshot of what information is generally available.

## Conclusions

Australian parents, when searching for immunisation information are largely finding technically correct information which isn’t well attributed, without explanatory depth. Conversely there are some sites which provide well-referenced highly technical information, which is challenging for the general public to understand when assessed against usual health literacy conventions. Furthermore, this information is often not easily identified in the initial stages of searching. Our findings suggest a need for information which is easily found, transparently authored, well-referenced, and written in such a way that highly technical information is conveyed in an easily understood format informed by an understanding of parents’ needs and cognitions associated with interpreting health information.

## Abbreviations

GAVCS: Global advisory committee of vaccine safety
WHO: World health organization
